# Ceftriaxone-Induced Leukopenia During Inpatient Rehabilitation: A Case Report

**DOI:** 10.7759/cureus.107807

**Published:** 2026-04-27

**Authors:** Luis D Rivera-Amador, Jean P Moliere, Olu Oyesanmi, Edwardo Ramos, Rigoberto Nuñez

**Affiliations:** 1 Transitional Year Medicine, HCA Florida Oak Hill Hospital, Brooksville, USA; 2 Physical Medicine and Rehabilitation, University of Puerto Rico, Medical Sciences Campus, Puerto Rico, USA; 3 Internal Medicine, HCA Florida Blake Hospital, Bradenton, USA; 4 Physical Medicine and Rehabilitation, HCA Florida Blake Hospital, Bradenton, USA

**Keywords:** adverse drug reaction, ceftriaxone, leukopenia, neutropenia, rehabilitation

## Abstract

Ceftriaxone is a commonly used third-generation cephalosporin with a favorable safety profile and widespread use across acute and post-acute care settings. Although rare, hematologic adverse effects such as leukopenia and neutropenia have been reported. These complications may be underrecognized in inpatient rehabilitation populations, where patients frequently have multiple comorbidities and ongoing antimicrobial exposure.

An 80-year-old man undergoing inpatient rehabilitation for critical illness myopathy developed progressive leukopenia following initiation of ceftriaxone for a suspected urinary tract infection. Baseline laboratory studies demonstrated anemia and thrombocytopenia with a normal white blood cell (WBC) count. After ceftriaxone initiation, the WBC count declined to a nadir of 2.73 ×10³/µL with an absolute neutrophil count (ANC) of 1.45 ×10³/µL. The patient remained clinically stable without infectious complications.

The temporal relationship between ceftriaxone exposure and leukopenia, improvement following drug discontinuation, and absence of alternative etiologies support a diagnosis of probable ceftriaxone-induced leukopenia. Beta-lactam-associated leukopenia is typically idiosyncratic, reversible, and more likely to occur in older adults and patients with comorbid illness. In rehabilitation settings, unrecognized cytopenias may disrupt therapy participation and delay discharge planning.

Ceftriaxone-induced leukopenia is an uncommon but clinically significant adverse effect that may occur even during short courses of therapy. Routine laboratory monitoring and early recognition are essential in inpatient rehabilitation settings to prevent complications and ensure continuity of functional recovery.

## Introduction

Ceftriaxone is a third-generation beta-lactam cephalosporin widely used in both acute and post-acute care settings due to its broad antimicrobial spectrum, favorable pharmacokinetics, and convenient once-daily dosing. It is commonly prescribed for respiratory tract infections, urinary tract infections, intra-abdominal infections, and bloodstream infections, particularly among older adults with multiple medical comorbidities [1]. These characteristics contribute to its frequent continuation during transitions from acute hospitalization to inpatient rehabilitation.

Although ceftriaxone is generally well tolerated, hematologic adverse effects, including leukopenia, neutropenia, thrombocytopenia, and, in severe cases, agranulocytosis, have been reported [1,2]. Non-chemotherapy drug-induced leukopenia is typically idiosyncratic, unpredictable, and reversible following drug discontinuation. Antibiotics represent one of the most frequently implicated medication classes in drug-induced agranulocytosis, with beta-lactam antibiotics consistently identified as common culprits [2,3].

The pathophysiology of ceftriaxone-associated leukopenia is not fully understood but is thought to involve immune-mediated mechanisms, direct suppression of granulopoiesis within the bone marrow, or a combination of both. Drug-dependent antibodies targeting circulating neutrophils have been described, as well as reversible bone marrow suppression affecting myeloid precursors [3-5]. These reactions are typically delayed, occurring after several days to weeks of exposure, supporting an immune- or marrow-mediated process rather than acute toxicity [6].

Advanced age, prolonged antibiotic exposure, and multiple medical comorbidities have been identified as risk factors for beta-lactam-associated neutropenia [6,7]. These features are common among patients admitted to inpatient rehabilitation facilities, where individuals frequently present with residual effects of critical illness, baseline cytopenias, nutritional deficiencies, and ongoing antimicrobial therapy initiated during acute hospitalization. Despite this risk profile, laboratory monitoring practices in rehabilitation settings may be less standardized than in acute care hospitals.

Leukopenia in rehabilitation patients carries clinical implications beyond increased susceptibility to infection. The development of cytopenias may prompt changes in antimicrobial therapy, additional diagnostic evaluation, or temporary modifications in rehabilitation intensity, potentially delaying functional recovery and discharge planning. Early recognition of medication-induced leukopenia is therefore critical to ensuring patient safety while maintaining continuity of rehabilitative care.

Because ceftriaxone-induced leukopenia is uncommon, the existing literature primarily consists of case reports, small case series, and pharmacovigilance analyses. Continued reporting of such cases is essential to improving clinician awareness, refining risk stratification, and informing monitoring strategies. This case contributes to the literature by highlighting ceftriaxone-associated leukopenia occurring in an inpatient rehabilitation setting after a relatively short treatment course.

## Case presentation

An 80-year-old right-handed male with a past medical history significant for obstructive sleep apnea, hypertension, atrial fibrillation, hypothyroidism, and heart failure with preserved ejection fraction was admitted to an inpatient rehabilitation facility following hospitalization for critical illness myopathy. The patient had longstanding comorbid conditions; however, the exact duration of each condition was not consistently documented in the available medical record.

At the time of admission (hospital day 2), laboratory evaluation demonstrated normocytic normochromic anemia and thrombocytopenia, with a normal white blood cell (WBC) count of 5.40 × 10³/µL. Baseline cytopenias were noted, although no prior history of leukopenia was documented. Renal and hepatic function were within acceptable limits. The patient’s home medication list was reviewed and did not include agents commonly associated with bone marrow suppression. No new medications known to cause myelosuppression were initiated prior to the onset of leukopenia.

On hospital day 7, the patient developed dysuria, and a urinary tract infection was clinically suspected based on presenting symptoms; however, urine culture confirmation was not definitively documented, representing a limitation in establishing the indication for antibiotic therapy. Ceftriaxone 2 g daily was initiated empirically for a presumed complicated urinary tract infection. At that time, the WBC count was 4.06 × 10³/µL.

**Figure 1:**
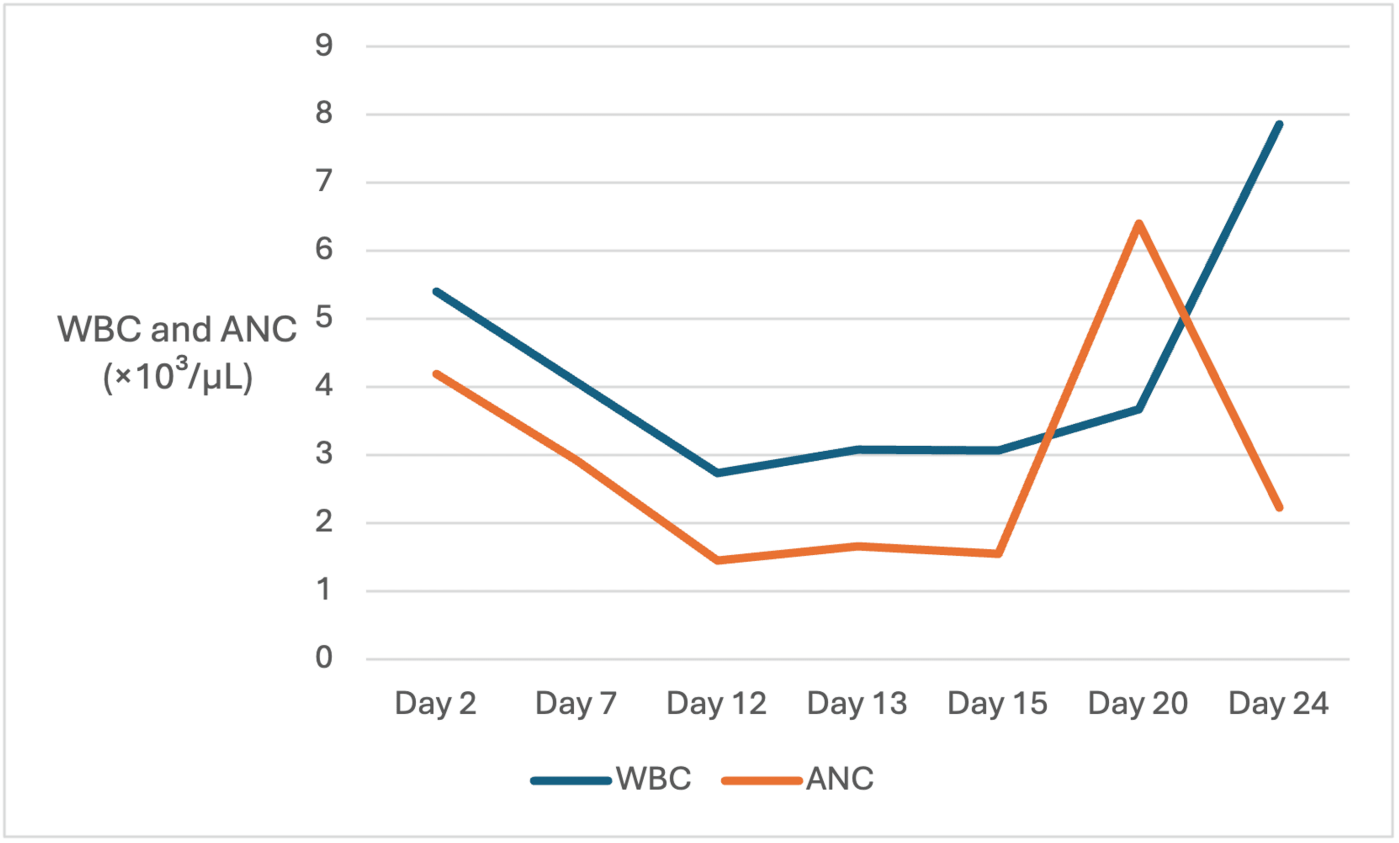
Trends in white blood cell (WBC) and absolute neutrophil count (ANC) during hospitalization Both WBC and ANC declined following the initiation of ceftriaxone on hospital day 7, reached a nadir on day 12, and subsequently recovered after discontinuation on day 15. These changes occurred in the context of a relatively stable baseline anemia and thrombocytopenia.

In the days following initiation of ceftriaxone, the WBC count progressively declined, reaching 2.73 × 10³/µL on hospital day 12. The absolute neutrophil count (ANC) concurrently decreased to 1.45 ×10³/µL, consistent with mild neutropenia. Leukopenia persisted on subsequent laboratory testing, with WBC values of 3.08 ×10³/µL on hospital day 13 and 3.06 ×10³/µL on hospital day 15. Following initiation of ceftriaxone, both the WBC count and ANC demonstrated a progressive decline, reaching a nadir on hospital day 12. In contrast, anemia and thrombocytopenia were present at baseline and remained relatively stable throughout the hospitalization. These trends are further illustrated in Figure 1, which demonstrates the temporal relationship between ceftriaxone exposure and changes in WBC and ANC.

The laboratory values corresponding to these trends are presented in Table 1, which provides detailed quantitative data for WBC count, ANC, and other hematologic parameters throughout the hospitalization. In addition to leukopenia, Table 1 demonstrates persistent anemia and thrombocytopenia, which remained relatively stable compared to the more dynamic changes observed in WBC and ANC.

**Table 1:** Laboratory trends during hospitalization Reference ranges: WBC, 4.0–11.0 ×10³/µL; absolute neutrophil count (ANC), 1.5–8.0 ×10³/µL. Bold values indicate nadir measurements. The ANC decreased to 1.45 ×10³/µL, consistent with mild neutropenia. The data demonstrate a progressive decline in WBC count and ANC following the initiation of ceftriaxone, with a nadir observed on hospital day 12 and recovery after discontinuation. Baseline anemia and thrombocytopenia were present and remained relatively stable throughout the clinical course. Abs: absolute

Laboratory Test	Reference Range	Day 2	Day 7	Day 12	Day 13	Day 15	Day 20	Day 24
White Blood Cell (WBC; ×10³/µL)	4.0 – 11.0	5.40	4.06	2.73	3.08	3.06	3.67	7.86
Hemoglobin (g/dL)	13.0 – 17.0	9.4	9.0	9.1	9.2	9.3	9.8	9.6
Hematocrit (%)	40 – 52	28.5	26.5	27.5	27.2	28.0	28.8	29.2
Platelets (×10³/µL)	150 – 400	143.0	116.0	102.0	113.0	122.0	106.0	95.0
Immature Granulocytes Abs (×10³/µL)	0.00 – 0.03	0.01	0.01	0.01	0.01	0.01	0.04	0.00
Neutrophils Abs (×10³/µL)	1.5 – 8.0	4.19	2.92	1.45	1.66	1.54	6.40	2.23
Lymphocytes Abs (×10³/µL)	1.0 – 3.0	0.71	0.56	0.68	0.76	0.90	0.71	0.94
Monocytes Abs (×10³/µL)	0.2 – 0.8	0.32	0.45	0.36	0.37	0.38	0.65	0.37
Eosinophils Abs (×10³/µL)	0.0 – 0.5	0.15	0.10	0.21	0.27	0.22	0.04	0.11
Basophils Abs (×10³/µL)	0.0 – 0.1	0.03	0.03	0.03	0.02	0.02	0.02	0.02

Alternative causes of cytopenia were considered during the clinical course. The patient’s medication list was reviewed, and no additional agents known to cause myelosuppression were identified. There was no clinical history suggestive of autoimmune disease, and no prior episodes of leukopenia were documented. Nutritional deficiencies (e.g., vitamin B12 or folate deficiency) were considered; however, there were no clinical features suggestive of severe deficiency, and the temporal pattern of leukocyte decline was not typical of nutritional cytopenias. Infectious causes, including viral etiologies, were considered less likely given the absence of systemic symptoms and the patient’s clinical stability. While formal diagnostic testing for some of these conditions was not performed, the overall clinical picture supported a likely drug-related process.

Given concern for ceftriaxone-induced leukopenia, the antibiotic was discontinued on hospital day 15 and replaced with amoxicillin-clavulanate. Following discontinuation, the patient’s WBC count demonstrated gradual improvement, increasing to 3.67 ×10³/µL by hospital day 20 and normalizing to 7.86 ×10³/µL by hospital day 24. The patient remained clinically stable throughout hospitalization and was discharged home with continued functional improvement.

## Discussion

Drug-induced leukopenia and neutropenia are uncommon but clinically significant adverse events that require careful evaluation, particularly in medically complex patients [2,3]. Antibiotics, especially beta-lactams, are among the most frequently implicated non-chemotherapy agents, although these reactions remain rare and often idiosyncratic [2,8,9,10]. In this case, the temporal association between ceftriaxone initiation and the subsequent decline in WBC count, followed by recovery after discontinuation, suggests a possible drug-related effect. However, this interpretation must be considered in the context of important clinical confounders.

A key limitation in attributing causality in this case is the presence of baseline hematologic abnormalities. The patient demonstrated anemia and thrombocytopenia prior to ceftriaxone exposure, consistent with underlying bone marrow vulnerability in the setting of recent critical illness. Although the WBC count was within normal limits at baseline, the coexistence of multilineage cytopenias complicates the distinction between drug-induced leukopenia and other potential contributors, such as critical illness-related marrow suppression, systemic inflammation, or nutritional deficiencies [3,5].

Alternative etiologies for leukopenia were considered. Infection-related marrow suppression was deemed less likely given the absence of systemic signs of infection and the patient’s clinical stability; however, microbiologic confirmation of urinary tract infection was not documented, which limits certainty regarding the indication for antibiotic therapy. Viral causes were not strongly suspected based on the clinical presentation, although specific viral testing was not performed and therefore cannot be definitively excluded. Nutritional deficiencies and autoimmune etiologies were also considered; however, the clinical presentation and temporal pattern of decline and recovery were not typical of these conditions. Importantly, no other medications known to cause myelosuppression were identified. The absence of microbiologic confirmation of infection and incomplete exclusion of all alternative etiologies further limit the strength of causal inference.

Despite these competing considerations, several features support ceftriaxone as a likely contributing factor. The decline in WBC and ANC occurred after initiation of therapy, reached a nadir during continued exposure, and improved following discontinuation. This clinical course is consistent with prior reports of beta-lactam-associated neutropenia, which typically develops after several days of exposure and resolves following withdrawal of the offending agent [6,11]. Proposed mechanisms include immune-mediated neutrophil destruction and reversible suppression of granulopoiesis [3,4]. Nonetheless, the observed association does not establish definitive causality. As a single case report, this observation should be interpreted cautiously and cannot establish definitive causality.

The Naranjo adverse drug reaction probability scale yielded a score consistent with a “probable” drug reaction; however, this tool has recognized limitations in complex clinical scenarios with multiple confounding factors [12,13]. Accordingly, it should be interpreted as supportive rather than definitive evidence, and clinical judgment remains essential.

From a rehabilitation perspective, leukopenia may have implications beyond infection risk, including potential interruptions in therapy and delays in discharge planning. Advanced age, comorbid illness, and ongoing antimicrobial exposure are recognized risk factors for beta-lactam-associated neutropenia and were present in this case [6,7].

Overall, this case highlights the diagnostic challenges of attributing leukopenia in medically complex patients. While ceftriaxone appears to be a plausible and likely contributor, the presence of baseline cytopenias, recent critical illness, and incomplete exclusion of alternative etiologies necessitates a cautious interpretation. Routine laboratory monitoring and early recognition of potential medication-related cytopenias are essential in similar clinical settings.

## Conclusions

Ceftriaxone-induced leukopenia is an uncommon but clinically relevant adverse effect that may occur even during short courses of therapy. In this case, ceftriaxone was a plausible and likely contributing factor; however, causality cannot be definitively established given the presence of baseline cytopenias and recent critical illness. Clinicians should maintain awareness of this potential complication and consider routine hematologic monitoring, particularly in high-risk patients, to support timely recognition and management.
